# Ethical Questions Raised by AI-Supported Mentoring in Higher Education

**DOI:** 10.3389/frai.2021.624050

**Published:** 2021-04-30

**Authors:** Laura Köbis, Caroline Mehner

**Affiliations:** Institut für Bildungswissenschaften, Professur für Allgemeine Pädagogik, Leipzig University, Leipzig, Germany

**Keywords:** AI, ethics, mentoring, higher education, AIED

## Abstract

Mentoring is a highly personal and individual process, in which mentees take advantage of expertise and experience to expand their knowledge and to achieve individual goals. The emerging use of AI in mentoring processes in higher education not only necessitates the adherence to applicable laws and regulations (e.g., relating to data protection and non-discrimination) but further requires a thorough understanding of ethical norms, guidelines, and unresolved issues (e.g., integrity of data, safety, and security of systems, and confidentiality, avoiding bias, insuring trust in and transparency of algorithms). Mentoring in Higher Education requires one of the highest degrees of trust, openness, and social–emotional support, as much is at the stake for mentees, especially their academic attainment, career options, and future life choices. However, ethical compromises seem to be common when digital systems are introduced, and the underlying ethical questions in AI-supported mentoring are still insufficiently addressed in research, development, and application. One of the challenges is to strive for privacy and data economy on the one hand, while Big Data is the prerequisite of AI-supported environments on the other hand. How can ethical norms and general guidelines of AIED be respected in complex digital mentoring processes? This article strives to start a discourse on the relevant ethical questions and in this way raise awareness for the ethical development and use of future data-driven, AI-supported mentoring environments in higher education.

## Introduction

Mentoring is widely accepted as a very beneficial, personal, and individual support, in which mentees take advantage of expertise and experience to expand their knowledge and to achieve individual goals. However, the emerging use of AI in mentoring processes in higher education not only necessitates the adherence to applicable laws and regulations (e.g., relating to data protection and non-discrimination) but also further requires a thorough understanding of ethical norms, guidelines, and unresolved issues (e.g., integrity of data, safety and security of systems, as well as confidentiality, avoiding bias, insuring trust, and transparency of algorithms).

The current boom of AI is accompanied by new institutes, initiatives, university courses, books, and papers which deal with the topics of tech ethics and AI ethics (Hagendorff, 2020). However, research, guidelines, and policies are still lacking for areas of application such as Artificial Intelligence in Education (AIED) (Holmes et al., 2019), and not all specific use cases have yet been taken into consideration. In this article, we want to address the missing ethical principles of AI-supported mentoring to start a discourse on this special application of AIED. To do so, we compare ethical principles of face-to-face mentoring with those of AI from the literature and start a discourse on ethical questions raised by AI-supported mentoring.

To begin with, the *Related Work: Mentoring Ethics and Artificial Intelligence Ethics* section will give an overview about the recent discourse in the fields of mentoring ethics and AI ethics. Subsequently, the *Ethical Principles and Questions of Artificial Intelligence-Supported Mentoring in Higher Education* section will connect these two fields and establish ethical principles of AI-supported mentoring and raising ethical questions that derived from one use case in higher education. Final conclusions and future work will be discussed in the last section. Regarding the key terminology, first, for the purposes of this article, our definition of AI will be provided in the *Artificial Intelligence Ethics* section. Second, we define AI-supported mentoring as a digitally enhanced and scalable process involving the typical topics of a mentoring relationship as follows: career support, support with expert knowledge, and socio-emotional support. In such processes, AI tools take over and automate mentors’ responsibilities, such as giving individual feedback or personal recommendations, helping to design individual learning paths or career strategies, all on the basis of the collected and analyzed data.^1^ This leaves us with a broad range of AI technologies that could be useful for AI-supported mentoring. Third, with regard to ethics, whether descriptive or normative, ethical norms describe and situate action in social values (e.g., Bartneck et al., 2019, 23–27). Although not directly consequential, ethical norms have a relationship with law, often acting as deputy or inspiration for non-existing statutory or case law, and play an important role in influencing soft law (ibid., 29).

## Related Work: Mentoring Ethics and AI Ethics

In this section, we will first outline the ethical questions raised and the guidelines stipulated in the context of face-to-face mentoring in recent literature. Second, we will describe the ethical concerns and guidelines regarding the increasing application of AI.

### Mentoring Ethics

Despite the lack of a consistent definition of mentoring, numerous articles and books have emphasized the positive impact mentoring can have on individuals by enabling them to benefit from the knowledge of a more-experienced individual in various contexts, for example, in sports, at work, or in education (Kram, 1985; see also; Johnson and Nelson, 1999; Petersen et al., 2017). Specifying the ethical questions of mentoring necessitates a clear understanding what mentoring is (for a good overview of definitions, see Ziegler, 2009). One of the definitory disputes is whether to include “bad mentoring,” or whether a mentor is “necessarily good in the way a hero or saint is necessarily virtuous” (Weil, 2001). In any case, there are more and more institutionalized programs that call themselves “mentoring programs,” so regardless of nomenclature, there is a practical need to evaluate relationships and to guide participants. Therefore, for the purposes of this article, we adopt a broad concept of mentoring that can include certain relationships otherwise referred to as coaching, counseling, or one-to-one teaching.

While conventional mentoring relationships are often relatively intimate and therefore not easy to observe (Weil, 2001), dysfunctions and issues such as harassment, mistreatment, exclusion, incompetence, deception, micromanagement, favoritism, or the reinforcement of power and hierarchy negatively affecting mentoring relationships have been identified (Moberg and Velazquez, 2004). Thus, the necessity to clarify the ethical questions and to develop guidelines for mentors and mentees to promote the quality of mentoring relationships and to avoid harm has driven scholarly inquiries across domains. Here, we introduce three prominent approaches, from the disciplines most prolific, with regard to mentoring ethics: a) business ethics, b) psychology, and c) education.

a) In their article, “The Ethics of Mentoring,” Moberg and Velazquez (2004) develop a set of ethical responsibilities of mentors. They argue that mentors have more responsibilities in the mentoring relationship than mentees, because they assume a “quasi-professional role”; provide “the protégé with the benefits of knowledge, wisdom, and developmental support”; and typically wield “superior power” (ibid.). They propose a model based on utilitarian principles (maximizing the good and minimizing harm), rights principles (taking into consideration individuals’ rights to be treated as a free and rational person), additional principles of justice (equitizing the distribution of benefit and burden), and principles of caring (even arguing for a certain degree of “legitimate” partiality). Accordingly, these four ethical principles imply the following seven mentor obligations:

**1. Beneficence:** to do good, specifically to provide knowledge, wisdom, and developmental support to mentees
**2. Nonmaleficence:** to avoid harming mentees through the exercise of power
**3. Autonomy:** to inform mentees about all the actions that one undertakes on their behalf and to ask for their consent
**4. Confidentiality:** to keep information about mentees’ confidential, to respect the mentees’ right of privacy, and to give them control about their information
**5. Fairness:** to avoid discrimination and to ensure that benefits and potential burdens to mentees are fairly distributed
**6. Loyalty:** to avoid conflicts of interests
**7. Concern:** to exercise a caring but fair partiality toward mentees and their interests
List 1: Mentors’ Obligations I

Moberg and Velazquez (2004) point out that these seven obligations may conflict with other moral obligations as well as with themselves and they suggest in such cases to act after the obligation, which is more “stringent.” The principle “concern” has, in our opinion, a huge conflict potential. It seems debatable whether a partiality towards a mentee can be fair and ethical or whether it rather means to harm and discriminate against others who have no access to this mentorship, and in this way reinforce power, hierarchy, and cronyism. Furthermore, from our point of view, the principles of “beneficence” and “nonmaleficence” are closely related and must not necessarily be seen as different principles.

b) In comparison to Moberg and Velazquez’ business ethics perspective, the second discipline home to numerous works on mentoring ethics is psychology. Johnson (2003) proposes a model to evaluate and conceptualize mentor competence and suggests nine principles that mentors in the field of psychology should adhere to (Johnson, in Palmer 2019):

**1. Beneficence:** promote mentees’ best interests whenever possible.
**2. Nonmaleficence:** avoid harm to mentees (neglect, abandonment, exploitation, and boundary violations).
**3. Autonomy:** work to strengthen mentee independence and maturity.
**4. Fidelity:** keep promises and remain loyal to those you mentor.
**5. Justice:** ensure fair and equitable treatment of all mentees (regardless of cultural differences).
**6. Transparency:** encourage transparency and open communication regarding expectations.
**7. Boundaries:** avoid potentially harmful multiple roles with mentees and discuss overlapping roles to minimize risk for exploitation or bad outcomes.
**8. Privacy:** protect information shared in confidence by a mentee and discuss all exceptions to privacy.
**9. Competence:** establish and continue developing competence.
List 2: Mentors’ Obligations II

Just as Moberg and Velazquez (2004), Johnson emphasizes the ethical principles of “beneficence,” “nonmaleficence,” and “autonomy” but uses different terms for the principles “loyalty” (Johnson calls it “fidelity”), “fairness” (Johnson refers to it as “justice”), and “confidentiality” (in Johnson’s list “privacy”). Interestingly, he adds the principles of “transparency” regarding the mentees’ and mentors’ “expectations,” “boundaries,” and “competence.” One could compare and interpret that Moberg and Velazquez (2004) include the principle “competence” in the principle “beneficence,” as they explain that the mentor needs to provide knowledge, wisdom, and support to the best he or she is able to. The ethical principle “boundaries” could be seen as part of the principle “nonmaleficence,” but specifically focuses on the harm of overlapping roles.

The APA’s Ethical Principles of Psychologists and Code of Conduct “can be seen as an aspirational guide to appropriate interactions with others in many situations, including mentoring.” It is subdivided into five general principles and 10 standards (American Psychological Association, 2002). In the APA’s guide for mentors and mentees, the general principles are applied to the mentoring relationship. In contrast to Johnson and Moberg and Velazquez (2004), APA emphasizes that mentoring is a mutual and reciprocal relationship, in which the mentee as well as the mentor can benefit from the other. Therefore, the APA’s principles are addressing both the mentor and the mentee and need to be taken into consideration from both of them:

**1. Principle A, Beneficence and Nonmaleficence:** Mentors and mentees should try to help others and be careful not to harm them. Both help and harm in the mentoring relationship may be hard to define and will depend on the purpose and context of the mentoring relationship.
**2. Principle B, Fidelity and Responsibility:** Leads the mentor to clarify the roles of each party to the relationship (the mentor will help the mentee and not just use the mentee to further his or her own career). The roles may evolve over time, and mentees and mentors should be aware of the possible changes in both roles and responsibilities.
**3. Principle C, Integrity:** Follows from the previous principle. Both the mentor and mentee need to do what they have agreed to do when establishing the relationship. If a point of conflict or confusion arises, each person should be willing to resolve that issue.
**4. Principle D, Justice:** Calls mentors and mentees to aspire to fairness, and to ensure that access is free from inappropriate bias. By virtue of Principle D, psychologists consider choices they make regarding with whom they will enter a mentoring relationship, and explore their reasons for choosing a particular mentee as opposed to other possible individuals who may desire such a relationship.
**5. Principle E, Respect for People's Rights and Dignity:** Guides both the mentor and mentee to consider personal differences so that any differences do not bias their interactions. This principle also serves as a reminder that in some mentoring relationships, there may be a power differential that could impact the process.
List 3: Mentors’ and Mentees’ Obligations Based on the APA’s Guide for Mentors and Mentees (summarized), 2002

APA’s principles give a broader overview of potential ethical problems, but sum up similar issues as Moberg and Velazquez (2004) and Johnson. In the APA’s guide for mentors and mentees^2^, it is emphasized that “both parties should have clear expectations of what the professional relationship can do and what it should not do” and that training and personal reflection can foster a successful and growth-oriented relationship (ibid.).

c) Finally, to include a general education perspective, we want to state the principles of the German Federal Association “Forum Mentoring e.V.” (Brückner, 2014), a nation-wide platform for research and networking around the topic, “Mentoring in academia.” Its praxis-oriented compendium establishes quality standards for mentoring in higher education, which try to define conceptual prerequisites, institutional frameworks and structure and elements of mentoring programs.^3^ With regard to the relationship between a mentee and a mentor, the following seven characteristics are emphasized (ibid.):

**1. Voluntariness:** mentee and mentor take part voluntarily in the mentoring program
**2. Independence:** mentee and mentor are not in a direct dependent relationship, for example, PHD supervisor and PHD student
**3. Defined time span:** the relationship evolves over a defined program time, but may be carried forward afterward
**4. Personal contact:** face-to-face contact is important for the success of the relationship, and may be supplemented by e-mail, chat, video calls, etc.
**5. Confidentiality:** mentoring conversations take place in a safe environment, and all shared information is confidential
**6. Commitment and liability:** mentor and mentee comply with agreements and appointments
**7. Expectations and agreements:** mentor and mentee communicate about concrete expectations and agreements.
List 4: Prerequisites for a Mentoring Relationship Based on Forum Mentoring e.V. (Summarized and Translated)

“Forum Mentoring e.V.” adopts a more practically oriented approach and the standards refer to institutionalized mentoring programs. Therefore, the recommendations for a mentoring relationship differ from the aforementioned ethical mentoring principles but also focus on “confidentiality,” “commitment,” “liability,” and “independence.” Some other general conditions of the relationship are mentioned, including “personal contact,” “defined time span,” and “voluntariness” that we would not necessarily include as ethical principles.

After discussing and comparing principles regarding mentoring ethics in three disciplines, we will now move on to Artificial Intelligence to approach the question, “which ethical principles need to be added or eliminated when we use AI-supported mentoring?”

### AI Ethics

Before focusing on ethical principles concerning AI, we have to precise the scope of AI in this article. We adopt the definition provided by the Stanford Encyclopedia of Philosophy, a source widely regarded as authoritative in the field of ethics. Accordingly, AI is “any kind of artificial computational system that shows intelligent behaviour, i.e., complex behaviour that is conducive to reaching goals. […] we incorporate a range of machines, including those in ‘technical AI’, that show only limited abilities in learning or reasoning but excel at the automation of particular tasks, as well as machines in ‘general AI’ that aim to create a generally intelligent agent” (Müller, 2020). This leaves us with a broad range of different AI-supported technologies and use cases, which makes it difficult to pin down all the possible ethical implications. Nevertheless, AI ethics, the young field within applied ethics (ibid.), suggests policy recommendations, which we want to refer to. Main debates evolve around privacy, surveillance, manipulation, opacity of AI systems, human–robot interaction, automation and employment, autonomous systems, machine ethics, artificial moral agents, and singularity (ibid.). Ethical questions concerning these aspects arise with certain uses of AI technologies, and do not arise with others (ibid.).

Before referring to general AI ethics principles, we want to focus on data ethics as an example. It is not only the data itself, its collection and the selection of appropriate data that raise ethical questions, but also its subsequent use (Zweig, 2019: 75–76). To focus on the dataset itself, the first question that needs to be addressed is how to gain access to data. To get a deeper insight, we can use historical data. Historical data can provide a basis for the deeper understanding and findings of events in the past, for the construction of hypotheses, and for setting a ground for future predictions (McCarthy et al., 2019: 11). In addition to or to cover a gap of missing historical data, new data will be collected to access a wider range of the field. In this context, a second question is arising, “how do we get access to a wide (and representative) range of data?” In addition, we have to consider first which kind of data we have access to. Afterward, we have to examine whether to focus on internal, self-produced and easily accessible data or how to get access to additional external data that can help support to raise the representativity of the dataset (Finlay, 2014).

Within all proceedings, the third question of representativity is very sensitive. The problem of algorithmic biases already occurred in the choice of representatives: [...] we will seek for a sufficient presence, a consistent treatment, and a proper representation of items, particularly those belonging to protected or disadvantaged groups (Castillo, 2019). The representation of disadvantaged groups and underrepresentation of minorities on the one hand is opposed by the problem of the construction of reality by predominant ringleaders. Activity data, in particular, are affected by what Baeza-Yates (2016) calls Zipf’s Law of minimal effort. That is, most people do not generate content, but they just watch the content generated by others. This implies that a minority of active users generates more than half of the content and hence the wisdom of the crowds is really the wisdom of a few. Danks and London (2017) provide a taxonomy of types and sources of algorithmic biases and give practical examples for 1) training data bias, 2) algorithmic focus bias, 3) algorithmic processing bias, 4) transfer context bias, and 5) interpretation bias. They argue to differentiate between problematic biases and neutral or even beneficial biases (ibid.). We now have an idea of what might be problematic regarding the access of datasets. In the next step, its automatic processing in an AI is encapsulated in what Zweig (2019) calls the OMA principle: the Operationalization (or Measurement, which we already discussed), the aspect of Modeling which means its abstraction and the application of principles. The A in OMA stands for the algorithm itself that needs to be focused on and which can strongly be influenced by algorithmic biases (referring to the process of access to data and its simplification). The intransparency in the process lead to what Müller (2020) points out as problem of opacity of AI systems: “With these techniques, the ‘learning’ captures patterns in the data and these are labelled in a way that appears useful to the decision the system makes, while the programmer does not really know which patterns in the data the system has used. In fact, the programs are evolving, so when new data comes in, or new feedback is given (‘this was correct’, ‘this was incorrect’), the patterns used by the learning system change. What this means is that the outcome is not transparent to the user or programmers: it is opaque.”

The questions arising in this paragraph frame reflections on AI ethics, as they address the basic problems of representativity and fairness, and they already occur before the processing of data in AI. It is widespread common sense that AI-supported technologies should be used for a common good. AI-supported technologies should not be used to harm or undermine anyone, and should respect widely held values such as fairness, privacy, and autonomy (Cramer et al., 2018). The amount of publications and standardizations in the field of ethical AI in general is continuously emerging. In this article, we want to exemplify three (international) resources concerning ethical principles as follows: a) The IEEE publication on “Ethically Aligned Design,” b) “The Ethics Guidelines for Trustworthy Artificial Intelligence” by the European Commission, and c) a report handed in by the German Commission on Data Ethics instituted by the German Federal Government.^4^

a) The IEEE Global Initiative on Ethics of Autonomous and Intelligent Systems published a list of eight principles for a fair, transparent, and human AI, that have been updated in 2019. Their perspective addresses developers in the field of AI and names eight aspects to consider within its development:

1. **Human Rights:** A/IS shall be created and operated to respect, promote, and protect internationally recognized human rights.
2. **Well-being:** A/IS creators shall adopt increased human well-being as a primary success criterion for development.
3. **Data Agency**: A/IS creators shall empower individuals with the ability to access and securely share their data, to maintain people’s capacity to have control over their identity.
4. **Effectiveness:** A/IS creators and operators shall provide evidence of the effectiveness and fitness for the purpose of A/IS.
5. **Transparency:** The basis of a particular A/IS decision should always be discoverable.
6. **Accountability:** A/IS shall be created and operated to provide an unambiguous rationale for all the decisions made.
7. **Awareness of Misuse:** A/IS creators shall guard against all potential misuses and risks of A/IS in operation.
8. **Competence:** A/IS creators shall specify and operators shall adhere to the knowledge and skill required for safe and effective operation.

List 5: Principles According to IEEEs “Ethically Aligned Design,” 2019.

Interesting in this perspective is the practical view on the realization and development, and the direct address of the stakeholders in the field. Especially, the aspects of “effectiveness,” “competence,” and “knowledge” refer directly to requirements of its realization.

b) A more political and juridical perspective is opened up with “The Ethics Guidelines for Trustworthy Artificial Intelligence” by the European Commission. They formulate guiding ethical values and refer to possible guidelines and legal references in the detailed description:

**1. Human agency and oversight:** including fundamental rights, human agency, and human oversight
**2. Technical robustness and safety:** including resilience to attack and security, fall back plan and general safety, accuracy, reliability, and reproducibility
**3. Privacy and data governance:** including respect for privacy, quality and integrity of data, and access to data
**4. Transparency**: including traceability, explainability, and communication
**5. Diversity, non-discrimination and fairness:** including the avoidance of unfair bias, accessibility and universal design, and stakeholder participation
**6. Societal and environmental well-being:** including sustainability and environmental friendliness, social impact, society, and democracy
**7. Accountability**: including auditability, minimization, and reporting of negative impact, trade-offs, and redress.
List 6: The Ethics Guidelines for Trustworthy Artificial Intelligence by the European Commission, 2019.

This more political position frames even the legal aspects of AI Ethics. They address the respect of fundamental rights and furthermore in this recommendation, especially aspects concerning data and “data governance,” refer directly to the already asserted rights in many European countries.

c) In the report of the German Commission on Data Ethics instated by the German Federal Government, general rights and principles on AI are communicated. The report then aligns these principles in fundamental, technical, and data referred aspects and specifies especially data processing in algorithmic systems. The following seven principles are displayed in the report and have been summarized and translated into English:

1. **Human Rights (Dignity):** human-centered design and respecting humans as a social being
2. **Self**-**Determination:** a manifestation of individual freedom, right to individual identity, and digital self-determination
3. **Privacy:** safety of human rights, dignity, and preserve integrity
4. **Safety:** safety of privacy, physical, and emotional security
5. **Democracy:** to guarantee human rights and to provide and secure education
6. **Fairness and Solidarity:** accessibility of (digital) resources
7. **Sustainability:** ecological, societal, and economical funding

List 7: Principles according to the report of the German Commission on Data Ethics, 2019.

As well as in the guidelines of the European Commission, in this ethics report, a political and legal view is emphasized and is closely related to the existing German law. Especially, the aspect of informational self-determination refers to aspects guaranteed in the German jurisdiction and the German law on data privacy and protection.

## Ethical Principles and Questions of AI-Supported Mentoring in Higher Education

In this section, we will compare the ethical principles of mentoring and AI evaluated in *Related Work: Mentoring Ethics and Artificial Intelligence Ethics* to start a discourse on the lacking principles of AI-supported mentoring. For the first time in this research context, we search for overlapping, redundant, and missing principles to suggest a basis for relevant ethical questions with the main aim to raise awareness for the ethical development, and use of future data-driven AI-supported mentoring environments in higher education.

### AI-Supported Mentoring Ethics

**FIGURE 1:**
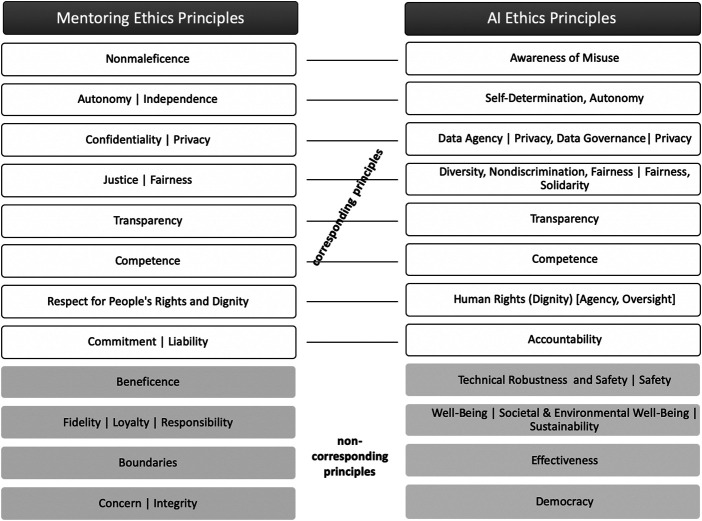
Juxtaposition of corresponding and non-corresponding ethical principles in the contexts of mentoring versus AI.

In Figure 1, corresponding and non-corresponding ethical principles of mentoring and AI are visualized. In spite of the limitations of the simplified representation of already condensed principles, the main benefit of the juxtaposition is the identification of unique and overlapping principles in both fields. Of course, we have to bear in mind that even within the disciplines, concepts and terminology differ, and both fields are very broad so that it is difficult to pin down all possible ethical implications. Furthermore, both domains are very distinct. While traditional face-to-face mentoring is highly personal and relationship based and ethical implications focus mainly on interpersonal relationships, AI ethics principles focus on the technology itself, human use of technology, and technology implications for society. Nevertheless, and interestingly, we found that the majority of principles and ideas on ethics are based on similar values.

In Figure 1, these corresponding principles are juxtaposed: in some cases, the correspondence is merely conceptual, for example, “Nonmaleficence” and “Awareness of Misuse,” where in both domains, the principle is based on the notion that people should not be harmed. In other cases, both concept and terminology are identical (e.g., “Fairness” and “Transparency”).

In contrast, we also identified principles that are unique in both contexts (highlighted in grey). Because of their focus on the personal relationship, the mentoring ethics principles, listed on the left, do not concentrate on the aspects of “technical robustness” and “safety,” “sustainability” and “equivalent aspects,” “effectiveness,” and “democracy.” However, we argue that traditional face-to-face mentoring can also benefit from considering AI ethics principles that raise more awareness of possible sensitivity of data, which is the basis for all mentoring relationships. The security aspects of the data that a mentee share with mentors go beyond “privacy” and “confidentiality” and can also lead to technical security issues in face-to-face mentorship (e.g., if mentors store notes or personal data of mentees on their PCs, mobile calendars, or other devices). Also, aspects like “democracy” and “sustainability” could be easily adapted to mentoring guidelines.

**FIGURE 2:**
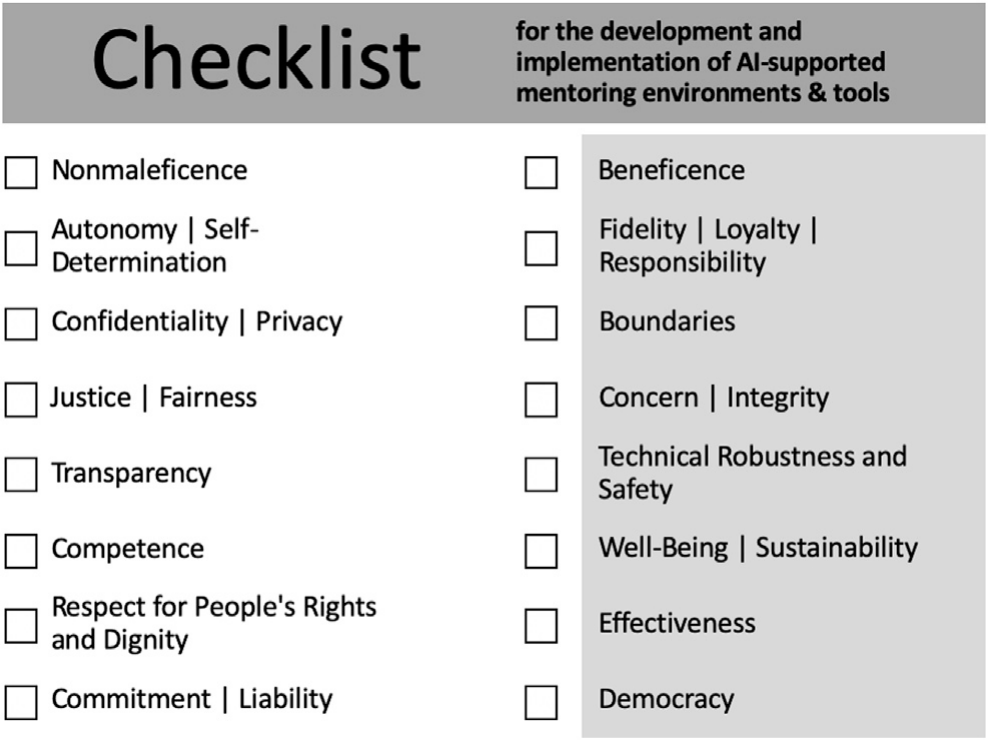
Checklist for the development and implementation of AI-supported mentoring environments and tools.

Reflecting upon our special use case of AI-supported mentoring, both AI ethics principles and mentoring ethics principles need to be considered. As it is an interdisciplinary topic, this comparison of principles can raise awareness of the ethical development and use of future data-driven, AI-supported mentoring environments in higher education. Interestingly, the non-corresponding principles (highlighted in blue) also play an important role and could easily be overlooked by stakeholders with only an educational/mentoring background or an IT/AI background. Therefore, we compiled a checklist for the responsible design of AI-supported mentoring environments and tools (Figure 2). As already mentioned, the range of technologies for AI-supported mentoring is huge (e.g., recommender systems for career paths, chat-bot mentors, or intelligent matching). Some ethical issues might be more relevant for certain AI-mentoring tools and less for others but have to be discussed and thought by the developers. In the *Exemplary Ethical Questions of the Use Case* section, we will focus on one exemplary use case to illustrate various ethical questions the checklist can raise. The aim of this study was not to answer these questions, but to raise awareness.

### Exemplary Ethical Questions of the Use Case

On the basis of the identified ethical principles of AI-supported mentoring, we would now like to highlight and discuss a few of them in an exemplary use case in the higher education context. The raised questions are only a starting point for a new discourse, and of course, many more questions and arguments are imaginable.

“… Suppose you are the head of the career service at a German University. Your university wants to establish a new professional AI-supported mentoring program that suggests students to apply for three job positions based on their information given in several online surveys and the learning data the system collected on their learning management system over the past semester.”

a) Would it be ethical to suggest three career options to the mentee without making transparent which of the collected data was used and how?

In this question, the center of interest is the principle of “transparency”: first, we have to ask, display and explain which data was collected. According to this we have to ask where the data was collected: which access points in the highly private mentoring process were set, and was there consent on the collection and processing of this data for the consideration of career options? Here, another facet according to the conception of AI-supported systems is affected; the “effectiveness,” which questions the purpose of the use of random data. Second, we should make transparent the representability of the data: whose data were used, which model was used, and what was the scope of the recommendation in the process? In this context, the principle of “fairness” needs to be respected.

b) Would it be ethical to include a passus in the data protection regulation that permits the university to use the students’ data also as training data for study program marketing purposes to guarantee long-term financial security of the mentoring program?

We already argued that mentoring requires one of the highest degrees of trust, openness, and socio-emotional support, as much is at stake for mentees, especially their academic attainment, career options, and future life choices. Highly personal data will be shared with the AI-supported mentoring program and not only the alignment with the university’s data protection regulations, but also the principles of “confidentiality and privacy” and “loyalty” need to be respected. If the program includes such a prerequisite in its data protection regulation, maybe students who do not agree will be excluded. However, the university is under constant financial pressure and in alignment with the principle “sustainability” must ensure that the program will run for future cohorts.

c) Would it be ethical to store the algorithm on an open server (e.g., GitHub) to save costs and provide a free program for all?

Here, some principles and reasons might oppose: we could come to the conclusion that the use of an open system and a good documentation for the further use and development of software affects “sustainability” and that the possibility to access code drives the “transparency” of its use. On the other side, an openly documented code might be an open door for technical intruders and a possibility to hack the system. Especially, when it comes to a sensitive set of personal data in the mentoring process, the system needs to guarantee a high standard of “technical robustness and safety.”

d) Would it be ethical to free students from the decision-making process behind the searching for positions on the job market they are interested in (e.g., going to job fairs, doing internships, talking to experts, and exploring their own talents and interests) and enhance their life choices by providing them tailored career paths?

On the one hand, this question relates to the mentoring principle “autonomy, independence, and self-determination”; a mentor should try to strengthen the mentee’s independence and maturity, and therefore, should not have such an enormous influence on the career and life of the mentee. On the other hand, AI support could rise above subjective recommendations of parents and teachers that students are exposed anyways. AI could be more objective and could show broader career opportunities. It is also imaginable that AI suggestions could ensure “fairness, justice, and diversity” because students who do not already have a social network that can provide them with internships and career support would also have access to the AI-supported mentoring program. With regard to the principle “competence,” Youyou et al. (2015) already demonstrated that personality judgments based on Facebook Likes are more accurate than those made by humans. However, it is important to consider that all recommendations and recommender systems can only be heuristics (Zweig, 2019).

These are only four of the many questions and discussion points that could be raised; of course, much more are imaginable. After considering these exemplary questions (and maybe already having more on your mind), would you like the idea of an AI-supported mentoring program? Would you, as head of the career service, introduce the program? Would you, as an educator, encourage your students to take part? Would you, as a student, rely on the suggested job positions?

## Discussion and Future Work

Technology today “raises fundamental ethical questions about community, democracy, and what it means to be human” (Michael Sandel in Ritchie, 2019). The use of AI in education can target behavioral change, involves very sensible and personal data, and thus, AI-supported pedagogical activities such as mentoring need to be “ethically warranted” (Holmes et al., 2019). It is therefore necessary to discuss ethical principles before developing, implementing, and using data-driven AI-supported mentoring environments in higher education.

In this article, we have juxtaposed principles of mentoring ethics and AI ethics with the objective of raising awareness in this interdisciplinary field. In our comparison, we have shown that many classical ethical questions have to be considered in both contexts. Despite the two different domains, we have identified numerous similar ethical principles. Interestingly, the non-corresponding principles of both domains also play an important role and could easily be overlooked by stakeholders with only an educational/mentoring background or a computer science/AI background. For this reason, ethical questions should be discussed interdisciplinarily. AIED guidelines and principles require the involvement of various stakeholders (students, philosophers, teachers, policymakers, parents, developers, etc.) (Holmes et al., 2019). We argue that it is the same in the context of AIME (AI-supported mentoring).

Both face-to-face mentoring and AI-supported mentoring bear advantages and disadvantages and ethical challenges: as long as AI cannot feel or interact with empathy and compassion, there will still be a great need for human teachers (Holmes et al., 2019) and mentors. However, the personal support of a human mentor can only reach a few beneficiaries, whilst AI-supported mentoring could democratize favoritism of the chosen elite. In addition, the new challenges that AI raises can also have a positive impact on classical face-to-face mentoring. The human–machine interaction demonstrates the vulnerability of the mentoring relationship and raises awareness of data security issues, data protection, biases in data, and data-driven mentoring support even in face-to-face situations.

The use of AI in mentoring environments creates new ethical challenges, but both face-to-face and AI mentoring will and can exist simultaneously, inspire one another and improve each other in a positive way. The juxtaposition of ethical principles in AI and mentoring in this article can serve as a basis and check list for further discussions in both disciplines and beyond. Following the idea of value-sensitive design (Friedman and Hendry, 2019), methods for a responsible design process of AI-supported mentoring technologies need to be developed in the future.

## Footnotes

1 As an example, we want to instance the digital mentor of the Politecnico di Milano, Graduate School of Business. Using the latest Microsoft AI tools, “Flexa'' analyzes hard, soft, and digital skills of participants, selects personalized content, helps with career and learning paths, as well as networking: https://www.som.polimi.it/en/flexa/. On their Web site, mentoring or AI ethics principles are not addressed.

2
https://www.apa.org/education/grad/mentoring

3
https://www.forum-mentoring.de/files/3014/1155/8650/FM_Broschre_A5Ansicht.pdf

4 For further insights in the field of ethical principles in AI, British researcher Alan Winfield carried together principles by numerous sources and has provided a deeper insight on his blog.

## References

[B25] American Psychological Association (2002). Ethical principles of psychologists and code of conduct. Available at: https://www.apa.org/ethics/code/ethics-code-2017.pdf (Accessed March 27, 2021).

[B1] Baeza-Yates R. (2016). “Data and algorithmic bias in the web,” in Proceedings of the 8th ACM conference on web science - WebSci’16, Hannover, Germany, May 22–25, 2016.

[B2] Bartneck C. Lütge C. Wagner A. R. Welsh S. (2019). Ethik in KI und robotik. München, Germany: Hanser.

[B4] Brückner S. (2014). Mentoring mit qualität. qualitätsstandards für mentoring in der wissenschaft. Forum mentoring. Available at: https://www.forum-mentoring.de/files/3014/1155/8650/FM_Broschre_A5Ansicht.pdf (Accessed March 27, 2021).

[B5] Castillo C. (2019). “Algorithmic bias in rankings,” in Companion proceedings of the 2019 world wide web conference (2019), San Francisco, CA, May, 2019. 10.1145/3308560.3316451

[B6] Cramer H. Garcia-Gathright J. Springer A. Reddy S. (2018). Assessing and addressing algorithmic bias in practice. Interactions 25, 6. 10.1145/3278156

[B7] Danks D. London A. J. (2017). “Algorithmic bias in autonomous systems,” in Proceedings of the 26th international joint conference on artificial intelligence (IJCAI), August, 2017, Sydney.

[B8] Finlay S. (2014). Predictive analytics, data mining and big data: myths, misconceptions and methods. Basingstoke, United Kingdom: Palgrave Macmillan.

[B9] Friedman B. Hendry D. G. (2019). Value sensitive design: shaping technology with moral imagination. Cambridge, United Kingdom: MIT Press.

[B10] Hagendorff T. (2020). The ethics of AI ethics: an evaluation of guidelines. Minds. Mach. 30, 99. 10.1007/s11023-020-09517-8

[B11] Holmes W. Bialik M. Fadel C. (2019). Artificial intelligence in education. Boston, MA: The Center for Curriculum Redesign Boston.

[B12] Johnson W. B. (2003). A framework for conceptualizing competence to mentor. Ethics Behav. 13, 2. 10.1207/s15327019eb1302_02

[B13] Johnson W. B. Nelson N. (1999). Mentor–protégé relationships in graduate training: some ethical concerns. Ethics Behav. 9, 3. 10.1207/s15327019eb0903_1

[B14] Kram K. E. (1985). Mentoring at work: developmental relationships in organizational life. Acad Manage J. 26, 4. 10.2307/2392687

[B15] McCarthy R. V. McCarthy M. M. Ceccucci W. Halawi L. (2019). Applying predictive analytics: finding value in data. Cham, Switzerland: Springer.

[B16] Moberg D. J. Velazquez M. (2004). The ethics of mentoring. Bus. Ethics Q. 14, 1. 10.5840/beq20041418

[B17] Müller V. C. (2020). “Ethics of artificial intelligence and robotics,” in The Stanford Encyclopedia of philosophy. Editor Zalta E. N.. Available at: https://plato.stanford.edu/archives/win2020/entries/ethics-ai/ (Accessed March 27, 2021).

[B18] Palmer C. (2019). How to mentor ethically. Mentoring the next generation of psychologists is one of the most important contributions you can make to the field. Here’s how to avoid ethical pitfalls while ensuring your mentees’ professional and academic success. Monitor Psychol. 50, 70.

[B19] Petersen R. Budde M. Brocke P. S. Doebert G. Rudack H. Wolf H. (2017). Praxishandbuch Mentoring in der Wissenschaft. Wiesbaden, Germany: Springer Fachmedien.

[B20] Ritchie R. (2019). Defining a new ethic for technology global intelligence for digital leaders. Available at: https://www.i-cio.com/management/insight/item/a-new-ethic-for-technology [Online]. (March 27, 2021).

[B21] Weil V. (2001). Mentoring: some ethical considerations. Sci. Eng. Ethics 7, 471–482. 10.1007/s11948-001-0004-z 11697003

[B22] Youyou W. Kosinski W. Stillwell D. (2015). Computer-based personality judgments are more accurate than those made by humans. PNAS 112, 4. 10.1073/pnas.1418680112 25583507 PMC4313801

[B23] Ziegler A. (2009). “Mentoring - konzeptuelle grundlagen und wirksamkeitsanalyse,” in Mentoring - theoretische hintergründe, empirische befunde und praktische anwendungen. Editor Stöger Lengerich D. H. (Lengerich, Germany; Pabst Science Publishers), 7–30.

[B24] Zweig K. A. (2019). Ein algorithmus hat kein taktgefühl, wo künstliche intelligenz sich irrt, warum uns das betrifft und was wir dagegen tun können. München, Germany: Heyne.

